# Tuberculosis in Men and Animals

**Published:** 1882-04

**Authors:** Thomas E. Satterthwaite


### THE JOURNAL

OF

### COMPARATIVE MEDICINE AND SURGERY.

VOL. III. APRIL, 1882. No. 2.

### ORIGINAL COMMUNICATIONS.

### ART. VI.—TUBERCULOSIS IN MEN AND ANIMALS: A STUDY IN COMPARATIVE PATHOLOGY.

BY THOMAS E. SATTERTHWAITE, M.D.

THERE are some practical questions relating to tubercle, or to the disease that commonly passes by that name, that have lately assumed unusual prominence in the public mind. One of them has arisen through the discussions in the public prints on vaccination, and we have been reminded from a certain quarter* that while the human family is being ostensibly protected against the scourge of small-pox, it is at the same time undergoing "physical deterioration" by being inoculated with other diseases that we know are extremely dangerous, if not deadly, in their effects upon the system. Then, again, the subject which the late Prof. Gerlach,† of Berlin, championed with so much activity has not been allowed to remain at rest, and we are told that the milk of tuberculous cows will produce tuberculosis in those who take it as food; and, not least of all, Toussaint is the authority who claims that the ingestions of raw meats and muscular juices, such as have become fashionable

* *Bergh.* The Lancet and the Law; North American Review, Feb., 1882, p. 161.

*Vidal.* Monthly Rev. of Med. and Pharm., Feb., 1882.

† *Gerlach.* Virchow's Archiv., 51, p. 290.

remedies for sickly children and invalids, is quite capable of initiating the same disease, if they have been derived from tuberculous sources.

It is proposed in the current article to review the subject of tuberculosis in such a way as to give a brief resumé of the various and, it may be said, alternating opinions that have been advanced during the many years it has been under discussion. In this way, the misconceptions and diversities of opinions that have so long prevailed will be better appreciated, and the reader will be gradually prepared to survey the ground upon which the matter stands at the present moment.

Among the early writers upon pulmonary consumption (for tuberculosis always enters into and forms part of one's ideas of consumption) there was an extreme latitude in the use made of the term tubercle. Bayle,* who is always referred to as one of the pioneers in this department, even named cancer as a form of tubercle. But, even after Laënnec's careful and masterly studies † had eliminated this and many other errors made by his predecessors, and given a rational if not a thoroughly satisfactory explanation of the pathological processes, we should not now have made much real progress towards the final solution of the matter, had not Virchow‡ still further narrowed the field of inquiry by rejecting from Laënnec's classification three out of four of that author's varieties of tubercle and all his tubercular infiltrations, and insisted that one form only, the miliary or the gray granulation, should be retained to represent the real tubercle of future scientific discussions

This was a great step forward, and this dogma has been recognized and respected up to the present day. Subsequently, histological studies (Niemeyer §) demonstrated that the infiltrated tubercle of Laënnec was more like a pneumonia, and it came to be thought that this *cheesy pneumonia* of phthisis was the frequent result of ordinary pneumonic inflammations. These ideas, however, were destined to receive a check from the researches of Von Buhl,|| who claimed to have proved by experiments upon animals that miliary tu-

---

* *G. L. Bayle.* Recherches sur la Phthisie Pulm naire. Paris: 1810, p. 18.

† *R. T. H. Laennec.* A Treatise on the Diseases of the Chest. Translated by John Forbes, M.D. New York: 1830, p. 278, *et seq.*

‡ *R. Virchow.* Die Krankhaften Geschwülte, II.

§ *Felix Von Niemeyer.* Clinical Lectures on Pulmonary Phthisis. Translated by J. L. Parke. New York: 1868.

*Ludwig V. Buhl.* Inflammation of the Lungs, etc. Translated by Drs. Mann and St. John. New York: 1874.

bercles were secondary products, resulting from the conveyance of irritating particles from distant foci. Since this time, more than ever, the solution of the question has been sought through animal experimentation; but here, too, errors were frequently committed, because the previous condition of the animal and its individual susceptibility had not been sufficiently studied, for animals that are often tuberculous under confinement, were used in experiment, and thus the value of the results became extremely problematical. And so it has happened, from this and other causes, that, though many hundreds—and perhaps thousands—of animals were utilized in the inquiry, the results obtained were for a long time little calculated to establish conviction in the mind; because, though clothed in some sort of a scientific dress, they were not conducted under the rigorous methods that alone can bring such questions to a final determintion.

Thus, while frogs are prone to miliary tuberculosis, the carnivora are not—even those that like dogs, live on a mixed diet. On the other hand, rodents—such as rabbits and guinea-pigs—are quite susceptible, while the ruminants, and especially those of the bovine race, have a remarkable tendency in this direction. And yet even these general statements have to be modified in some instances. Thus while animals of the bovine race exhibit tuberculosis with great frequency in confinement, they are not so apt to when at large. Then again age enters into the problem; for, as in the man family, the tendency to miliary tuberculosis of the lungs is great in adults, while in infants the membranes of the brain and the intestinal tract are the points of election, so in some of the lower animals the site of the disease is largely governed by the age of the individual.

A more critical review of the historical part of this question leads us to the fact that pulmonary phthisis (consumption) in some form or other has been recognized from very early times. It was even alluded to by Hippocrates,* but while his clinical account of the disorder was sufficient to distinguish it, his pathological notions were vague and absurd. Galen † also recognized and wrote of the disease, but it seems more just to regard Bonet‡ as one of the first who described

* *Hippocrates.* Œuvres Completes, T. VII. Littré, 1851, pp. 77, 189, 208.

† *Galen.* Epitome Galeni Operum. De Methodo Medendi, Lib. V., 422, 423, 1643.

‡ *Bonet.* Sepulchretum Sive Anatomia Practica, Lib. II., De Respiratione Læsa, Obs. 19, 1679.

tubercle with any degree of precision. Certainly in the edition of 1700, by Mangetus, tubercles receive a pretty careful mention, and he even appears to have recognized the miliary bodies. Virchow,* however, associates this fact with the name of Baillie.† Tubercular phthisis seems to have been first described by Bayle,‡ who regarded it as a specific disease, to be distinguished from other forms of phthisis that he called melanotic, ulcerous, calculous, granular and cancerous. Of more than 900 cases enumerated by him, 624 were of the tubercular variety, and 183 of the granular. Laënnec,§ however, was the first to give a sharp and intelligible delineation of the morbid appearances, and the fame of his writings and teachings secured for the views a wide acceptance. In 1819 he wrote that tuberculous matter was to be found under two forms, the one *insulated bodies* and the other *infiltrated substance*. Of the former there were four principal varieties, the *miliary*, the *crude*, the *granular*, and the *encysted;* the infiltrated substance was *irregular*, *gray*, or *yellow*. For him tuberculosis (pulmonary) was not inflammatory, and pneumonia was not transformed into phthisis. These views of Laënnec were subsequently sustained by Louis.‖ Having examined the records in M. Chomel's service at the Charité, where 67 phthisical patients died during a period of three years, he was enabled to say "nos observations confirment celles de M. Laënnec, et que, pour nous comme pour lui, l'existence des tubercules dans les poumons est la cause et constitue le caractère propre de la phthisie." Let us see if, with all the vast mass of work that has been done for more than half a century since that date, we have succeeded in changing the full force of each one of these remarkable words. Lebert,¶ who brought the microscope to bear upon the matter, still further corroborated the teachings of Laënnec and Louis, asserting confidently that both forms of tubercle already mentioned were of the same nature, containing the peculiar bodies that he called "tubercle corpuscles." Somewhat earlier, however, Rokitansky** gave in his adhesion to the same view, but called attention to the multi-nucleated

---

* *Virchow.* Die Krankhaften Geschwülste. Vol. II.

† *Matthiew Baillie.* The Morbid Anatomy, etc., 3d Amer., Phila., 1820.

‡ *G. L. Bayle.* Recherches sur la Phthisie Pulmonaire, Paris, 1809.

§Loc Cit.

‖ *P. C. A. Louis.* Recherches Anatomiques Pathologiques et Therapeutiques sur la Phthisie, Paris, 1825.

¶ *H. Lebert.* Traite Pratique des Maladies Scrofuleuses, etc., Paris, 1849, p.

** *Rokitansky.* Lehrbuch der Path. Anat. Wien., 1855, p. 289.

corpuscles (mutter-zellen), which he had observed in the tubercle granulum. It would appear that the first break in this solid array of opinion was made by Rheinhardt,* who showed that the so-called infiltrated tubercle, which, strangely enough, he held to be the most characteristic form of tubercle, was in reality an inflammatory exudation undergoing transformation. It was now that Virchow made the first grand move towards simplifying the problem, for he was able to substantiate Rheinhardt's views, and in so doing at once showed the folly of retaining the infiltrated tubercle as part and parcel of tuberculosis, which all regarded as non-inflammatory (Laënnec, Louis, Lebert and Rokitansky). If, said he (in substance) the name is retained, let it apply to the gray granulation of Laënnec or of Bayle. For even the yellow caseating tubercle, he argued, is confounded with other totally distinct lesions, such as inspissated pus and encysted parasites. This event proved, as we have already said, to be the commencement of a new era, and simplicity ruled where all before had been misunderstanding and doubt. From this time on, scientific pathologists, when speaking of tubercle, referred to the *miliary tubercle.* Naturally, as the result of the great pathologist's teachings, doubt was thrown upon many conditions that had previously been grouped with tuberculosis, such, especially, as laryngeal phthisis, while Niemeyer† even went so far as to connect pulmonary phthisis with pneumonia, of which it was a legitimate sequel.

According to him, the two prominent conditions under which pulmonary consumption was found were: First, chronic pneumonia; second, chronic pneumonia with secondary tuberculosis; third, primary tuberculosis. But Buhl,‡ of Munich, acting perhaps under ideas foreshadowed by Virchow, had formulated a theory in accordance with certain experiments on animals, and he appeared to prove that miliary tubercles were caused by the presence of one or more special sources of infection, that might be located in the vicinity or at some distance. Accordingly experiments on animals were made in great numbers, with the view of testing the truth or fallacy of this new doctrine and now the conclusions of Villemin,§ that had been previously made

* *Reinhardt.* Cellular Pathology, 1860, p, 159.

† *Niemeyer.* Lehrbuch der Spec. Path. u. Ther., 7te Aufl. I., p. 250, Berlin.
*Langhans* (Virchow's Archiv., 42, p. 382, 1868,) described them su sequently with some minuteness.

‡ *L. v. Buhl.* Loc Cit.

§ *J. A. Villemin.* Gaz. Med. de Paris, 1865, No. 50, p. 787. Etudes sur la Tuberbulose, etc. Paris, 1868.

public, but had met with little favor, came into prominence. Villemin, Professeur Agrègè at the Ecole Impériále of Val-de-Grace, had accumulated some experiences that he had been publishing since 1865. He used rabbits and guinea-pigs, and various other animals for his studies, inoculating them with the caseous matter of human phthisis, and his results appeared to prove conclusively that pulmonary tuberculosis (phthisis) is a disease specific in its nature, and communicable by inoculation, producing the well-known eruptions of miliary tubercles. Many of the animals so inoculated died, while others emaciated, and were then killed. The lesions were found to be located in the lungs chiefly, to a progressively lesser degree in the spleen, liver, bronchial and mesenteric glands, according to the order in which these several organs are named. In ruminants and small carnivora, inoculation usually produced no appreciable effect, but with horned cattle the results were positive, the animal (one case) dying in six weeks of emaciation, tubercles being found in the lungs, pleuræ, spleen, liver, kidneys, and mesenteric glands. Villemin, in reviewing this particular case, held, therefore, that bovine and human tuberculosis are identical, notwithstanding the now well-known fact that there is a morphological difference between the two, and a specific deposit in the former that appears to pathognomonic of the bovine disease.

The views of Villemin were not cordially received by the committee of the French Academy to which they were referred, but they served to stimulate a number of workers in various parts of the world, and especially such representative English investigators as Wilson Fox* and Burdon Sanderson,† who early opposed the views of the French writer, claiming that the effects produced were due chiefly to the introduction of an *irritant* into the system.

They adopted much the same methods as Villemin, and obtained results that were very analogous, certainly in the matter of cheesy products and tuberculous nodules; but they also found that the same results were obtained by inoculating animals with matter that was not tuberculous. In fact, they concluded that any *mechanical irritant* may do the same thing. Accordingly, the views advanced by Villemin, that tuberculosis is a specific disease, received a preëmptory

* *Wilson Fox.* Brit. Med. Journ., May 23—June 6, 1868.

† *Burdon Sanderson.* Med. Times and Gaz. 1868, p. 431.

check. Given, they said, any material that will produce a caseous deposit, no matter what that first cause may be, we then have a starting point from which miliary tubercles can be developed. These views were, therefore, in agreement with those of Buhl, and the chronological sequence of events was no longer deposition of gray tubercles, their aggregation, softening, caseation, and, subsequently, caseous infiltrations of the lung, but caseation at some point in the body, the lung, or anywhere else, then deposition of the gray bodies, and subsequently their aggregation, central softening, and, lastly, caseation.

This latter point was confirmed by Frænkel and Cohnheim,* who demonstrated on rabbits and guinea-pigs that any centre of suppuration in the body, at least in rabbits and guinea-pigs, will serve as the starting point for a general tubercularization. Such animals, therefore, are auto-inoculated, or, in other words, poisoned by their own secretions. These experiments had been repeated so many times that it seemed to be finally established that the order of phenomena was really as has already been indicated, and efforts were made to show in what various ways the same result may be accomplished, rather than to topple over a theory that had such a substantial basis in observed facts. In 1873, the great discussion on tuberculosis, calling out as it did an expression of opinion from those who had enjoyed the amplest experiences in Great Britain, was a further effort, and a successful one, which tended very largely to make the clinical distinctions of phthisis conform to the more modern pathological notions. In this discussion, Wilson Fox,† taking as a typical example the acute tuberculosis of children, found three sets of changes: first, the typical gray granulations; second, granulations composed of epithelial products, with growths in the walls of the vessels; third, large areas of the lung similarly affected, but in which the vesicles were almost completely obliterated, and the capillary circulation nearly abolished. Tubercle, he thought, was not histologically so simple as it had been described. Tuberculosis and phthisis were one and the same thing, the differences in different cases being comprehensible by supposing that they were different stages of the same process. If he were making a classification it would be merely into two varieties, acute and chronic, and the term

* *Cohnheim and Frænkel.* Virchow's Arch. 45, p. 216.

† *Wilson Fox.* Trans. Path. Soc. of London, 1873: Vol. xxiv, p. 307.

tuberculosis or phthisis might then be used according to individual preference.

Thus one readily sees how thoughtful studies of the pathological sequence of events may explain in a simple way the observed train of clinical phenomena.

Moxon * of London, on the same memorable occasion, claimed that pulmonary tuberculosis should not be regarded as a specific disease, i. e., in the sense advocated by Villemin, but rather as a special phase of inflammatory action. He associated tubercle with all forms of active phthisis, basing his views upon an examination of more than 500 bodies. There was but one exception, and this was when the disease had progressed slowly for a very long time; then the lung fell into the condition known as fibroid phthisis, and the caseous matters had been thrown off. In such a case, nature was operating to effect a cure. To him all phthisis was the tubercular phthisis of Laënnec and Louis, but broncho-pneumonia had no connection with it.

Let us now for a moment advert to the anatomical components of the miliary tubercle, and observe that as some ten years ago we were in the habit of picking out a gray granulation, tearing it to pieces under the microscope, and demonstrating the adenoid tissue, with its accompanying small corpuscular elements, as the essential features, and then in later years bending our attention to the giant cells, which were with great pains, if at all, dug out of the interior, then seeking with equal anxiety for Schüppel's epithelial bodies; † so now it is the fashion to emphasize another symptom, and this is central caseation, which appears to be uniformly present and readily recognized by the eye. ‡

Now, in order to reconcile these different microscopic appearances in accordance with the idea that they are all proper constituents of a tubercle granulum, it will be necessary to again retrace our steps a little.

(*To be continued in next number.*)

---

* *Moxon.* Trans. of the Path. Soc. of London, loc cit.

† *Schüppel.* Virchow's Archiv., 56, 1872.

‡ Some observers have not given in their adhesions to the simple ideas that have been indicated as offering a logical and satisfactory explanation of the pathologico-clinical history of pulmonary phthisis, but prefer to classify it in agreement with special microscopic findings.

*Petroff.* Virchow's Archiv., 44, b. 129.

